# Understanding acceptance of contactless monitoring technology in home-based dementia care: a cross-sectional survey study among informal caregivers

**DOI:** 10.3389/fdgth.2023.1257009

**Published:** 2023-10-04

**Authors:** Christian Wrede, Annemarie Braakman-Jansen, Lisette van Gemert-Pijnen

**Affiliations:** Centre for eHealth and Wellbeing Research, Department of Psychology, Health & Technology, University of Twente, Enschede, Netherlands

**Keywords:** remote monitoring, assistive technology, dementia, informal care, technology acceptance, implementation

## Abstract

**Background:**

There is a growing interest to support home-based dementia care via contactless monitoring (CM) technologies which do not require any body contact, and allow informal caregivers to remotely monitor the health and safety of people with dementia (PwD). However, sustainable implementation of CM technologies requires a better understanding of informal caregivers' acceptance. This study aimed to examine the (1) general acceptance of CM technology for home-based dementia care, (2) acceptance of different sensor types and use scenarios, and (3) differences between accepters and refusers of CM technology.

**Method:**

A cross-sectional online survey was conducted among *n* = 304 informal caregivers of community-dwelling PwD [Mean(SD) age = 58.5 (10.7)] in the Netherlands and Germany. The survey contained a textual and graphical introduction to CM technologies, as well as questions targeting (1) general acceptance of CM technology, (2) acceptance of seven different contactless sensor types, (3) acceptance of five different use scenarios, and (4) caregivers' own and their care recipients' personal characteristics. Data were examined using descriptive and bivariate analyses.

**Results:**

Participants' general acceptance of CM technology was slightly positive. We found significant differences in acceptability between contactless sensor types (*p* < .001). RF-based sensors (e.g., radar) and light sensors were considered most acceptable, whereas camera-based sensors and audio sensors (e.g., microphones, smart speakers) were seen as least acceptable for home-based dementia care. Furthermore, participants' acceptance of different use scenarios for CM technology varied significantly (*p* < .001). The intention to use CM technology was highest for detecting emergencies (e.g., falls, wandering), and lowest for predicting acute situations (e.g., fall prediction). Lastly, accepters and refusers of CM technology significantly differed regarding gender (*p* = .010), their relation with the PwD (*p* = .003), eHealth literacy (*p* = .025), personal innovativeness (*p* < .001), usage of safety technology (*p* = .002), and the PwD's type of cognitive impairment (*p* = .035) and housing situation (*p* = .023).

**Conclusion:**

Our findings can inform the development and implementation of acceptable CM technology to support home-based dementia care. Specifically, we show which sensor types and use scenarios should be prioritized from the informal caregiver's view. Additionally, our study highlights several personal characteristics associated with informal caregivers' acceptance of CM technology that should be taken into account during implementation.

## 1. Introduction

### 1.1. Background

Dementia is a leading cause of dependency in older people (1). The worldwide prevalence of dementia is expected to increase to 78 million by 2030 and 139 million by 2050 (1). In response to limited institutional care capacities (2), care reforms have shifted toward supporting people with dementia (PwD) to live at home for as long as possible (3, 4). While living at home with appropriate support is what most PwD desire (4), this often has a significant impact on their informal caregivers such as spouses, children, or other family members who bear the responsibility of caring for them, often from a distance (5–7).

As the burden on informal caregivers of community-dwelling PwD increases, so too does the need for innovative care solutions, including those from the field of assistive technology (8). The use of in-home monitoring technology enables informal caregivers to remotely monitor the lifestyle, health, and safety of PwD. Such technology allows informal caregivers a potentially greater sense of security and competence when managing care demands (9) and could help to delay institutionalization of PwD (10). Within this field, contactless monitoring (CM) technologies are of growing interest (11). CM technologies do not require any direct body contact or attachment to clothing, making them less prone to feasibility issues as compared to wearables (11, 12). Instead, sensors are placed in the living environment to capture relevant information. Different kinds of contactless sensors exist or are currently in development including, for example, audio sensors (e.g., microphones, smart speakers), visual sensors (cameras), smart energy meters (detecting energy consumption of household appliances), or object-tagged sensors (sensors attached to objects of daily use) (11–14). The most novel form of CM technology sensors (e.g., sensors using radar or Wifi channel state information) is based on the human body's reflection of radio waves to capture and model meaningful activities of daily living (15–18). While the technical maturity and implementation readiness of CM technology sensors differ (12), they all share the potential of moving from reactive to more proactive care at home for PwD and reduce caregiver burden (19).

However, despite their potential, future successful and sustainable implementation of CM technologies in home-based dementia care will, among other things, to a great extent depend on user acceptance (20). Informal caregivers may not wish to use all types of CM technologies in the care of their loved one with dementia. By taking factors that impede or promote the future acceptance of CM technology in home-based dementia care into account, it is possible to design and implement such technologies in a more acceptable way (21). Therefore, a good understanding of user acceptance of CM technology in home-based dementia care is warranted.

When studying user acceptance, different frameworks can be applied. Classical frameworks such as the technology acceptance model [TAM; (22)] and the unified theory of acceptance and use of technology [UTAUT; (23)] have been widely used. However, these frameworks have been criticized for being too generic and ignoring the context of use and characteristics of a technology (24, 25). This makes it difficult to draw conclusions for development and implementation of new technology (20). The CeHRes Roadmap (26), on the other hand, is designed to guide the development and implementation of health technology, making it easier to draw relevant recommendations for practice. The framework highlights the importance of achieving a good fit between the technology, use context, and users to ensure acceptance and successful implementation (26). Following this principle, developers of CM technology for home-based dementia care need to make adequate choices taking into account technological, contextual, and user-related aspects relevant for acceptance.

However, previous research has mainly focused on acceptance of technology in general among PwD and their caregivers (27–30) or has focused on healthy older adults (20, 24, 31, 32), consequently providing little guidance tailored to the context of CM technology for informal dementia care. First, there is still limited insight into informal caregivers' general acceptance of CM technology as well as towards technological options such as different contactless sensor types that could be used. Second, still little is known about informal caregivers' acceptance of different use contexts for CM technology. Our previous qualitative studies (33, 34) give reasons to believe that informal caregivers of community-dwelling PwD have diverse preferences to apply CM technology for different use scenarios. Lastly, there is still limited insight into individual differences in CM technology acceptance among informal caregivers of PwD, making it hard to apply tailored implementation strategies. Informal caregiver and care recipient characteristics have previously been reported to influence implementation of technology for informal caregivers (35, 36). This gives reasons to believe that CM technology acceptance might vary based on personal characteristics, especially given the fact that informal caregivers of community-dwelling PwD are a highly diverse group (35).

Taken together, an examination of the technological, contextual, and user-related aspects of acceptance of CM technology for home-based dementia care is needed to allow for better product development and implementation. To our knowledge, this current study is the first to focus on informal caregivers of PwD specifically and to apply a quantitative cross-sectional approach when doing so.

### 1.2. Aim of the study

The overarching aim of this study was to gain more insight into the acceptance of CM technology for home-based dementia care from the informal caregiver's perspective. More specifically, the study aims were:

(1) To explore the general acceptance of CM technology for home-based dementia care
(2) To explore the acceptance of different sensor types for CM technology
(3) To explore the acceptance of different use scenarios for CM technology
(4) To explore differences in personal characteristics between accepters and refusers of CM technology

## 2. Method

### 2.1. Study design

This study applied a quantitative cross-sectional design. Data were collected using an online survey among informal caregivers of community-dwelling PwD in the Netherlands and Germany.

### 2.2. Sample and procedure

Inclusion criteria for participation were as follows: Participants needed to be (1) 18 years of age or older and (2) voluntarily providing unpaid care to a community-dwelling person with dementia or mild cognitive impairment as main reason of care. Community-dwelling was defined as living at home or in any other non-institutional setting. Participants were acquired by nonprobability convenience sampling. Between May and December of 2022, participants were invited to take part in the study through different channels. Those included (a) mailing lists of caregivers from a previous study who participated in the evaluation of a digital care collaboration platform and indicated their interest in future research participation, (b) the websites of the Dutch and German Alzheimer Society, and c) different peer support groups for informal caregivers on Facebook (e.g., “Mantelzorg Dementie”, “Ik ben In voor Mantelzorg”). Before taking part in the study, participants provided informed consent on the first page of the online survey. Only completed questionnaires were used for further analysis.

### 2.3. Survey development

The online survey (see Supplementary Material) was developed by a multidisciplinary group consisting of researchers in the field of health technology development, and gerontology. Relevant topics and questions for the survey were formulated based on the research questions, prior research on stakeholders' attitudes and needs towards CM technology in home-based dementia care (33, 34), and research of others in the field of acceptance of technology for aging in place (21, 31). The survey was pre-tested among *n* = 3 older informal caregivers [Mean(SD)age = 71(0.8); *n* = 2 German and *n* = 1 Dutch] to collect feedback on the clarity of questions and instructions. Adjustments were made accordingly before the survey was set out for data collection. Approval for the study was obtained from the ethics committee of the University of Twente (Faculty BMS, request nr. 220793) according to European regulations.

### 2.4. Survey sections

In the following, the different components of the online survey are explained, listed in the same order in which they were presented to participants.

#### 2.4.1. Personal characteristics

Informal caregivers of community-dwelling PwD are a diverse group (3). To obtain meaningful information of informal caregivers' personal characteristics, our survey included questions that cover the typical characteristics that are used to profile this group of individuals (33). These questions included characteristics that have been found to influence implementation of technology for informal care (35, 36):

(1) Informal caregiver characteristics:
   - Single items asking for age, gender, relation with care recipient, size of informal care network, geographical distance to care recipient, and care technologies that informal caregivers already make use of
   - Validated questionnaires measuring perceived caregiver burden [4-item scale of the Zarit Burden Interview (ZBI-4) (37)], eHealth literacy [5-item subscale of the eHealth Literacy Questionnaire (eHLQ) focused on the ability to actively engage with digital services (38)], and personal innovativeness in the domain of information technology [4-item scale by Agarwal et al. (39)]. The scales for eHealth literacy and personal innovativeness were available in Dutch but not yet in German, and therefore translated via the procedure of translation and back translation (CW, MA) (40).
(2) Care recipient characteristics:
   - Single items asking for age, symptom duration, type of cognitive impairment, housing situation (living alone vs. living together), and usage of professional home care (yes/no).

#### 2.4.2. Introduction CM technology

To give participants an impression of CM technology for home-based dementia care, they were given a short textual introduction together with two illustrations depicting (a) the general concept of CM technology, and (b) a mock-up of a digital platform interface that visualizes possible monitoring information shared within the care network (see Supplementary Material).

#### 2.4.3. Acceptance of different sensor types

We operationalized acceptance of different sensor types as follows: Participants were asked to what extent they consider the use of different sensor types as acceptable in the care of their loved one with dementia, on a Likert scale from 1 (very unacceptable) to 5 (very acceptable). Based on recent reviews on sensor devices for human activity recognition (11, 12, 41), we included seven different contactless sensor types, categorized into either “device-free” sensors (stand-alone sensors placed into the living space), or “device-bound” sensors (sensors bound to devices in the living space). Device-free sensor types included: (1) RF-based sensors (e.g., radar), (2) audio sensors (e.g., microphones, smart speakers), (3) camera-based sensors (cameras that generate anonymized footage, i.e., images in which faces are not recognizable), (4) light sensors (sensors which detect whether the light is on or off), and (5) temperature/humidity sensors. Device-bound sensor types included: (1) object-tagged sensors (sensors attached to objects of daily use such as bed sensors, door sensors, or sensors attached to the fridge), and (2) energy meters (sensors that monitor energy consumption of household appliances).

#### 2.4.4. Acceptance of different use scenarios

Participants were presented with short descriptions of five different use scenarios for CM technology in home-based dementia care. Those represent the most relevant scenarios that have been distilled from our previous qualitative studies among key stakeholders for CM technology in home-based dementia care (33, 34):

(1) Detection of emergency situations (e.g., fall detection, wandering detection)
(2) Prediction of acute situations (e.g., fall prediction)
(3) Monitoring of selfcare behaviors (e.g., deviations in eating, drinking, washing, toilet use)
(4) Monitoring of nocturnal wellbeing (e.g., deviations from the usual sleeping pattern such as nocturnal unrest or a disturbed circadian rhythm)
(5) Monitoring of gradual health status changes (e.g., cognitive or physical changes within a certain period)

Different aspects of acceptance towards each of the scenarios were operationalized and measured as follows: (a) Intention to use [1 item: “I would like to use contactless monitoring technology for (scenario X) in the near future.”], (b) Acceptability for informal caregiver [1 item: “Contactless monitoring technology for (scenario X) is something that I would find acceptable.”], (c) Acceptability for care recipient [1 item: “Contactless monitoring technology for (scenario X) is something that my loved one would find acceptable.”], and (d) Perceived usefulness [6 items; example item: “Contactless monitoring technology for (scenario X) would be useful to me.”]. For all variables, participants answered on a Likert scale from 1 (totally disagree) to 5 (totally agree).

#### 2.4.5. General acceptance of CM technology

The final part of the survey assessed participants' general acceptance of CM technology, which was operationalized as follows: We measured intention to use CM technology by asking to what extent participants would agree to use CM technology in the care of their loved one with dementia for (a) “At this point in my life” and (b) “When cognitive or physical health of my care recipient declines”. Participants answered on a Likert scale from 1 (totally disagree) to 5 (totally agree).

### 2.5. Data analysis

The data were analyzed using IBM SPSS statistical software (version 25, IBM Corporation, Armonk, NY, USA). Descriptive statistics (means and standard deviations) were computed for (a) general acceptance of CM technology, (b) acceptance of different sensor types, (c) acceptance of different use scenarios (intention to use, acceptability, perceived usefulness), and (d) all continuous personal characteristics. Frequency distributions (*N*, %) were generated for all categorical personal characteristics.

Shapiro-Wilk tests were conducted on all continuous variables to test for normality. Since data were not normally distributed, nonparametric tests were used. To test for significant differences in general acceptance of CM technology between “At this point in my life” and “When cognitive or physical health of my care recipient declines”, a Wilcoxon test was performed. To test for significant differences in acceptance between sensor types, a Friedman test and post-hoc pairwise comparisons with Bonferroni correction were executed. In the same way, significant differences in acceptance (intention to use, acceptability, perceived usefulness) between use scenarios were tested.

To explore differences in personal characteristics between accepters and refusers, two steps were followed. First, we divided the sample into a) accepters, including those who “strongly agree” or “agree” to use CM technology in the care of their loved one at this point in their life (scores on general acceptance ≥4), and b) refusers, including those who “strongly disagree”, “disagree”, or are undecided to use CM technology in the care of their loved one at this point (scores on general acceptance ≤3). Subsequently, differences in personal characteristics between accepters and refusers of CM technology were tested by Mann–Whitney *U*-tests for continuous variables (age, caregiver burden, eHealth literacy, personal innovativeness) and Chi-square tests for categorical variables (gender, education, relation with care recipient, size informal care network, geographical distance to care recipient, usage of care technologies, symptom duration, type of cognitive impairment, housing situation, usage of professional home care). Statistical significance was set at *α* < .05.

## 3. Results

### 3.1. Sample characteristics

Upon invitation, *n* = 1,000 informal caregivers started the survey, of which *n* = 568 completed all questions. Of those, *n* = 304 met all inclusion criteria and were included in the analysis. Tables 1, 2 (column “Total”) show characteristics of the sample aged between 21 and 91 (*M* = 58.5; SD = 10.7). The majority of participants were female (*n* = 215, 71%), higher educated (*n* = 163, 54%), and were the adult children of a person with dementia (*n* = 189, 62%). Most participants cared for a person with Alzheimer's disease or vascular dementia (*n* = 161, 54%), who lives alone (*n* = 204, 67%). On average, the sample reported moderate caregiver burden (score 3 out of 5) and high eHealth literacy (score 3.2 out of 4). At the time of data collection, the majority of the sample neither used safety technology (e.g., alarm buttons, GPS trackers) (*n* = 180 non-users, 59%), digital communication technology (*n* = 169 non-users, 56%), nor technology to support memory/day structure (*n* = 227 non-users, 75%) in the care of their loved one with dementia. Most participants did, however, use digital care platforms to support the coordination of their loved one's care (*n* = 164 users, 54%).

**Table 1:** Differences in informal caregiver characteristics between accepters and refusers of CM technology.

Variable	Levels	Total (*n* = 304)	Accepters (*n* = 153)	Refusers (*n* = 151)	*P* ^c^
Age (mean, SD)		58.5 (10.7)	57.3 (10.9)	60 (10.5)	.077
Gender (*N*, %)	Female	215 (70.7)	98 (64.1)	117 (77.5)	.010*
	Male	89 (29.3)	55 (35.9)	34 (22.5)	
Country of residence (*N*, %)	Netherlands	225 (74)	112 (73.2)	113 (74.8)	.746
	Germany	79 (26)	41 (26.8)	38 (25.2)	
Education (*N*, %)^a^	University degree (Bachelor, Master, or higher)	163 (53.6)	87 (56.9)	76 (50.3)	.467
	Professional degree	64 (21.1)	27 (17.6)	37 (24.5)	
	High school diploma	72 (26)	37 (24.2)	35 (23.2)	
	Other	5 (1.6)	2 (1.3)	3 (2)	
Relation with care recipient (*N*, %)	Adult child (daughter/son)	189 (62.2)	85 (55.6)	104 (68.9)	.003*
	Spouse/partner	45 (14.8)	21 (13.7)	24 (15.9)	
	Daughter-/son-in-law	27 (8.9)	15 (9.8)	12 (7.9)	
	Sister/brother	6 (2)	3 (2)	3 (2)	
	Neighbor/friend	22 (7.2)	20 (13.1)	2 (1.3)	
	Other	15 (4.9)	9 (5.9)	6 (4)	
Size of informal care network (*N*, %)	1 person	98 (32.2)	47 (30.7)	51 (33.8)	.665
	2 persons	107 (35.2)	60 (39.2)	47 (31.1)	
	3 persons	56 (18.4)	27 (17.6)	29 (19.2)	
	4 persons	27 (8.9)	12 (7.8)	15 (9.9)	
	>5 persons	16 (5.3)	7 (4.6)	9 (6)	
Geographical distance to care recipient (*N*, %)	I live in the same house as the person I care for	55 (18.1)	24 (15.7)	31 (20.5)	.664
	1–5 min away	35 (11.5)	20 (13.1)	15 (9.9)	
	6–15 min away	70 (23)	33 (21.6)	37 (24.5)	
	16–30 min away	58 (19.1)	32 (20.9)	26 (17.2)	
	31 min-1 h away	43 (14.1)	24 (15.7)	19 (12.6)	
	>1 h away	43 (14.1)	20 (13.1)	23 (15.2)	
Caregiver burden (mean, SD)^b^		3 (0.6)	3.1 (0.8)	2.9 (0.8)	.141
eHealth literacy (mean, SD)^b^		3.2 (0.6)	3.3 (0.6)	3.1 (0.6)	.025*
Personal innovativeness (mean, SD)^b^		4.3 (0.8)	4.5 (0.8)	4.1 (0.8)	**<**.001*
Usage of care technologies (*N*, %)	Safety technology (e.g., alarm buttons, GPS trackers)				.002*
	User	124 (40.8)	76 (49.7)	48 (31.8)	
	Non-user	180 (59.2)	77 (50.3)	103 (68.2)	
	Digital communication technology (e.g., video calling, messaging apps)				.635
	User	135 (44.4)	70 (45.8)	65 (43)	
	Non-user	169 (55.6)	83 (54.2)	86 (57)	
	Technology to support memory/day structure (e.g., digital calendar, smart medicine dispenser)				.099
	User	77 (25.3)	45 (29.4)	32 (21.2)	
	Non-user	227 (74.7)	108 (70.6)	119 (78.8)	
	Digital care platforms to support the coordination of care				.426
	User	164 (53.9)	86 (56.2)	78 (51.7)	
	Non-user	140 (46.1)	67 (43.8)	73 (48.3)	

Significant *P*-values are shown in bold.

^a^ High school diploma = VMBO, HAVO, VWO (Netherlands); Haupt-/ Realschulabschluss, Abitur (Germany). Professional degree = MBO (Netherlands); Berufsschulabschluss (Germany).

^b^ Score ranges: Caregiver burden: 1–5; eHealth literacy: 1–4; personal innovativeness: 1–7.

^c^ Chi-square tests for categorical variables; Mann–Whitney *U*-tests for continuous variables.

**Table 2:** Differences in the characteristics of persons with dementia reported by accepters and refusers of CM technology.

Variable	Levels	Total (*n* = 304)	Accepters (*n* = 153)	Refusers (*n* = 151)	*P* ^a^
Age (mean, SD)		84.2 (8)	84.5 (8.1)	83.9 (7.7)	.673
Symptom duration (*N*, %)	<1 year	17 (5.6)	9 (5.9)	8 (5.3)	.742
	1–2 years	56 (18.4)	23 (15)	33 (21.9)	
	2–3 years	76 (25)	39 (25.5)	37 (24.5)	
	3–4 years	39 (12.8)	22 (14.4)	17 (11.3)	
	4–5 years	49 (16.1)	26 (17)	23 (15.2)	
	>5 years	67 (22)	34 (22.2)	33 (21.9)	
Type of cognitive impairment (*N*, %)	Alzheimer's disease	104 (34.2)	60 (39.2)	44 (29.1)	.035*
	Vascular dementia	57 (18.8)	25 (16.3)	32 (21.2)	
	Lewy Body dementia	8 (2.6)	6 (3.9)	2 (1.3)	
	Mild cognitive impairment (MCI)	45 (14.8)	16 (10.5)	29 (19.2)	
	Other dementia type	45 (14.8)	19 (12.4)	26 (17.2)	
	No official diagnosis yet	45 (14.8)	27 (17.6)	18 (11.9)	
Housing situation (*N*, %)	Living alone	204 (67.1)	112 (73.2)	92 (60.9)	.023*
	Living together	100 (32.9)	41 (26.8)	59 (39.1)	
Usage of professional home care (*N*, %)	Yes	206 (67.8)	110 (71.9)	96 (63.6)	.121
	No	98 (32.2)	43 (28.1)	55 (36.4)	

Significant *P*-values are shown in bold.

^a^ Chi-square tests for categorical variables; Mann–Whitney *U*-tests for continuous variables.

### 3.2. General acceptance of CM technology

**Figure 1:**
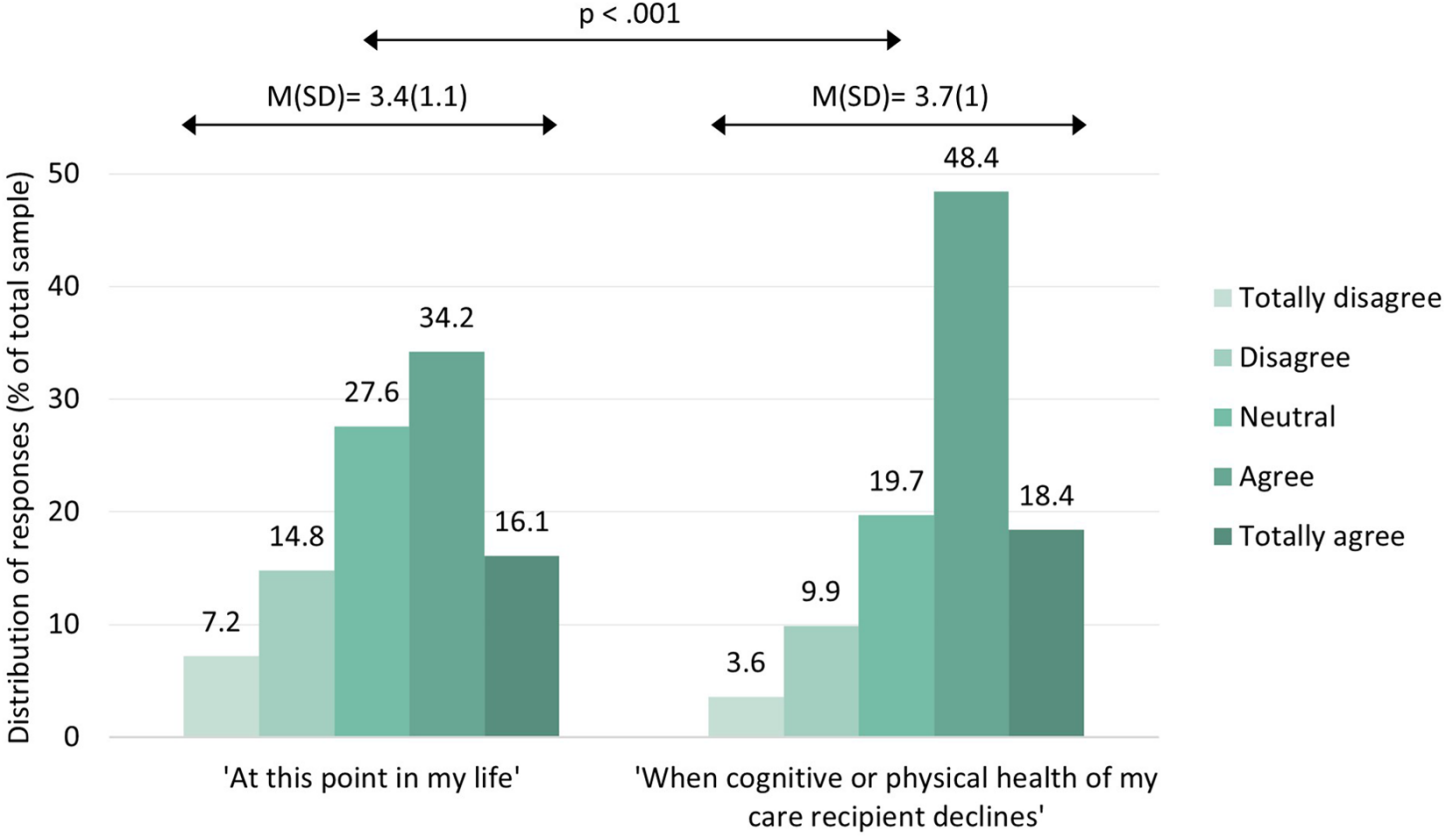
Intention to use CM technology for the current (left) and future care situation (right). Scores ranged from 1 (totally disagree) to 5 (totally agree).

Figure 1 shows that informal caregivers' general intention to use CM technology in the care of their loved one with dementia was, on average, slightly positive (mean score between 3 [= “neutral”] and 4 [= “agree”). Participants' intention to use CM technology was significantly higher for a future situation in which cognitive or physical health of their care recipient declines (*M* = 3.7; SD = 1) compared to at this point in their life (*M* = 3.4; SD = 1.1) (*Z* = −5.362, *p* < .001).

### 3.3. Acceptance of different sensor types

**Figure 2:**
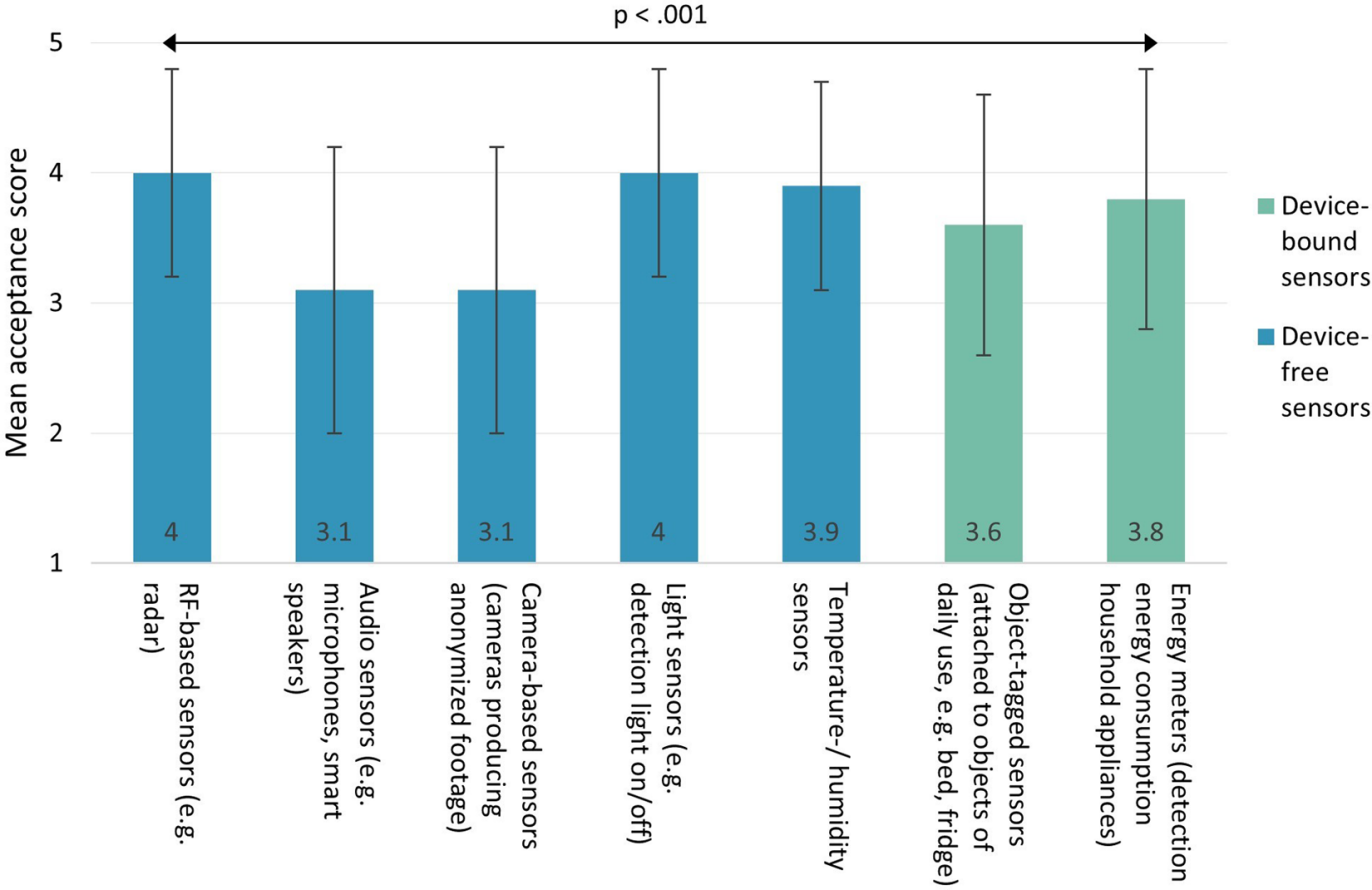
Acceptability of different contactless sensor types. Scores ranged from 1 (very unacceptable) to 5 (very acceptable).

Figure 2 shows informal caregivers' acceptance of different contactless sensor types, categorized into device-free (blue) and device-bound sensors (green). As can be seen, informal caregivers considered none of the seven sensor types as “very unacceptable” (score < 2) or “unacceptable” (score < 3) in the care of their loved one with dementia. On average, most acceptable sensor types included RF-based sensors (*M* = 4; SD = 0.8) and light sensors (*M* = 4; SD = 0.8), whereas least acceptable sensor types encompassed audio sensors (*M* = 3.1, SD = 1.1) and camera-based sensors (*M* = 3.1, SD = 1.1).

Inferential statistics revealed significant differences in acceptability between sensor types [*χ*^2^(6) = 272.71, *p* < .001]. Post-hoc pairwise comparisons showed that RF-based sensors, light sensors, temperature/humidity sensors, energy meters, and object-tagged sensors were all considered significantly more acceptable than audio sensors (*p* ≤ .001) and camera-based sensors (*p* < .001). Furthermore, object-tagged sensors were considered significantly less acceptable than RF-based sensors (*p* = .003) and light sensors (*p* < .001).

### 3.4. Acceptance of different use scenarios

**Figure 3:**
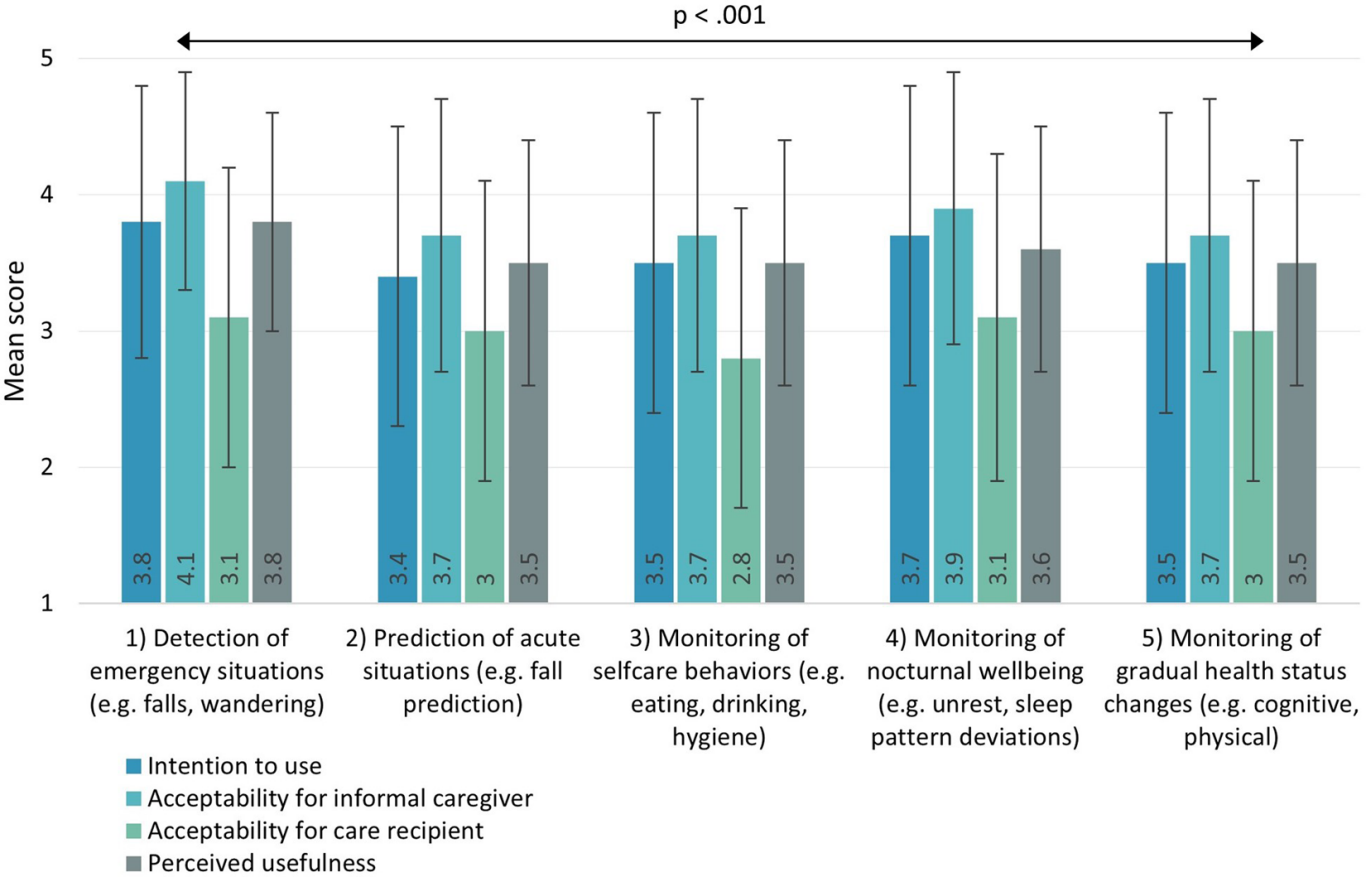
Intention to use, acceptability, and perceived usefulness for different use scenarios of CM technology in home-based dementia care. Scores ranged from 1 (totally disagree) to 5 (totally agree).

Figure 3 shows that, on average, none of the use scenarios received a score ≤2 (=“disagree”) on acceptability, perceived usefulness, and intention to use CM technology. Interestingly, informal caregivers viewed their own acceptability of CM technology for all use scenarios as higher than that of what they believed the acceptability of their loved one with dementia would be. Descriptive statistics furthermore show that intention to use CM technology was highest (*M* = 3.8; SD = 1) for scenario 1 (detection of emergency situations), and lowest (*M* = 3.4; SD = 1.1) for scenario 2 (prediction of acute situations).

Inferential statistics revealed significant differences between use scenarios in all tested variables (intention to use: *χ*^2^(4) = 63.329, *p* < .001; acceptability for informal caregiver: *χ*^2^(4) = 126.442, *p* < .001; acceptability for care recipient: *χ*^2^(4) = 54.185, *p* < .001; perceived usefulness: *χ*^2^(4) = 48.404, *p* < .001). Post-hoc pairwise comparisons for intention to use showed that participants' intention to use CM technology for scenario 1 (detection of emergency situations) was significantly higher than for most other scenarios, including scenario 3 (monitoring of selfcare behaviors) (*p* = .001), scenario 2 (prediction of acute situations) (*p* < .001), and scenario 5 (monitoring of gradual health status changes) (*p* = .032). Furthermore, intention to use CM technology for scenario 4 (monitoring of nocturnal wellbeing) was significantly higher than for scenario 3 (monitoring of selfcare behaviors) (*p* = .035) and scenario 2 (prediction of acute situations) (*p* = .008).

### 3.5. Differences in personal characteristics between accepters and refusers of CM technology

Tables 1, 2 provide an overview of significant differences in personal characteristics between accepters (*n* = 153) and refusers (*n* = 151) of CM technology for home-based dementia care. Table 1 presents the personal characteristics of informal caregivers whereas Table 2 presents those of their care recipients with dementia. Our analysis revealed the following:

Compared to refusers, accepters were significantly:

- more often male (*p* = .010),
- more often a neighbor or friend (*p* = .003) and less often an adult child (daughter/son) of the PwD (*p* = .003),
- more often caregiver of a PwD who lives alone (*p* = .023),
- less often caregiver of a person with mild cognitive impairment (*p* = .035),
- scoring higher on eHealth literacy (*p* = .025) and personal innovativeness (*p* < .001),
- more often users of safety technology (e.g., alarm buttons, GPS trackers) in the care of their loved one with dementia (*p* = .002)

Accepters and refusers did not significantly differ regarding age, country of residence, education, size of informal care network, geographical distance to care recipient, caregiver burden, age of care recipient, symptom duration of care recipient, and the care recipient's usage of professional home care.

## 4. Discussion

### 4.1. Principal findings

This study aimed to explore informal caregivers' acceptance of CM technology for home-based dementia care. We also examined caregivers' acceptance of different sensor types and use scenarios for CM technology, as well as differences in personal characteristics between accepters and refusers.

Our study showed that the general intention to use CM technology among Dutch and German informal caregivers of community-dwelling PwD was rather positive at this point in their life, and significantly higher for a future situation in which cognitive or physical health of their care recipient declines. At the same time, however, we found that accepters and refusers of CM technology did not significantly differ regarding their care recipient's symptom duration. Taken together, this indicates that an appropriate moment to offer CM technology can be at an early disease stage already, which is in line with our previous qualitative study among key stakeholders in home-based dementia care (34).

Our findings showed that none of the CM technology sensor types studied was considered unacceptable by informal caregivers of PwD, including camera-based sensors. This is surprising as in-home monitoring systems based on cameras often pose major challenges concerning privacy (11, 12). However, the example used in our study was cameras that produce anonymized footage (i.e., images in which faces are not recognizable), which might explain why participants had a rather neutral than negative opinion about it (42). Our participants considered RF-based sensors (e.g., radar) among the most acceptable sensor types for home-based dementia care. This indicates that the current shift in human activity recognition research towards device-free sensing based on analyzing the human body's reflection of radio waves (12) is in line with informal caregivers' expectations. However, additional research is needed to clarify underlying reasons for this finding (e.g., What made RF-based sensors more acceptable than others?). Based on our results, we recommend designers of CM technology for home-based dementia care to prioritize RF-based sensors, light sensors, temperature/humidity sensors, energy meters, and object-tagged sensors above audio sensors and camera-based sensors. However, it should be noted that sensor choice ultimately also depends on practical considerations. For example, RF-based sensors have the potential to continuously measure dynamic human activity, as opposed to object-tagged sensors which can only measure human-object interaction. On the other side, RF-based sensors are more prone to interference from the environment which can cause noise in the data (11). In addition, those sensors are able to measure through walls, which can be beneficial as sensors do not need to be placed in every room, but also problematic when data is collected unintentionally on uninvolved people outside the household (12).

Our study revealed that informal caregivers' acceptance of different use scenarios for CM technology varied significantly. Based on our results, we recommend that use scenarios such as “detection of emergency situations” (e.g., fall- and wandering detection) and “monitoring of nocturnal wellbeing” (e.g., nocturnal unrest, sleep pattern deviations) should be given priority during development and implementation of CM technology for home-based dementia care. We found the lowest intention to use CM technology for “prediction of acute situations” such as fall prediction. While there is a growing research direction within human activity recognition focused on algorithmic analytics to predict (fall) risk levels (11, 19), this does not seem to be a priority for informal caregivers in our study. It is possible that informal caregivers may have found it difficult to imagine how to act upon such risk predictions in terms of care- or household adjustments. Instead, this might rather be in the interest of care managers, as shown by a previous qualitative study among stakeholders for CM technology in home-based dementia care (34). Notably, informal caregivers viewed their own acceptability of all use scenarios as higher than that of what they believed the acceptability of their loved one with dementia would be. To better understand potential differences between both stakeholders, future research should obtain information from PwD directly instead of using proxy measures. In addition, shared decision-making tools, such as recently developed by Berridge et al. (43), may assist PwD and their caregivers to form a joint decision about the use of diverse care technologies at home, including monitoring technology.

Lastly, our study showed that accepters and refusers of CM technology for home-based dementia care significantly differed regarding gender, their relation with the care recipient, eHealth literacy, personal innovativeness, usage of safety technology, and their care recipient's type of cognitive impairment and housing situation. Interestingly, accepters of CM technology turned out to be significantly more often neighbors or friends, and significantly less often adult children of the PwD. This indicates that informal caregivers who do not have a family bond with the PwD might be more likely to accept CM technology. Adult children, on the other side, are often secondary caregivers in cases where the PwD lives together with a partner, which might reduce their need for CM technology. Additionally, privacy issues might make blood-relatives of PwD more reluctant about using CM technology in the home environment. It was also striking that accepters of CM technology were significantly more often users of safety technology (e.g., alarm buttons, GPS trackers) already in the care of their loved one with dementia. Previous research has shown that assistive technologies which are already used can prevent the acceptance of new technologies (24). However, this was not true for our participants, as they were more likely to accept CM technology if they used simple safety technology already. Interestingly, geographical distance to the PwD did not play a role in CM technology acceptance, suggesting that informal caregivers living 1 min away would be as much open for CM technology as those living more than 1 h away. All in all, those findings can help to inform implementation strategies for novel CM technology in home-based dementia care tailored to those who most likely intend to use the technology.

### 4.2. Limitations

Our study does not come without limitations. First, participants' responses were based on the provided survey materials and not on actual use of CM technology. This could have made it more difficult for participants to give their opinion about the technology. However, at the same time, it enabled us to focus on a broad range of possible use scenarios and sensor types at once, including novel RF-based sensor types which are still in development. Our previous qualitative research (33, 34) has furthermore shown that informal caregivers of community-dwelling PwD can form expectations towards CM technology based on textual and graphical explanations such as those used in the current survey.

Second, by using an online survey, informal caregivers of PwD with limited digital skills might have been less likely to respond. In fact, on average, our sample showed high eHealth literacy and the majority used digital platforms to support the coordination of their loved one's care. This might cause the sample to be less representative for current informal caregivers of community-dwelling PwD, but possibly more representative for potential future users of CM technology.

Lastly, due to the explorative character of this study, no multivariate analysis (binary logistic regression) was performed. Therefore, we cannot conclude which participant characteristics together act as predictors of intention to use CM technology.

### 4.3. Future research

Based on our study, several directions for future research can be formulated. First, our study has investigated acceptance of different sensor types and use scenarios of CM technology separately, whereas stated preferences towards combinations of those options can be even more insightful. To investigate this, future research could use a discrete choice experiment (DCE) design. In DCEs, participants make choices between different hypothetical “packages” of a product or service which systematically vary in certain attributes (44). The choices participants make can be used to infer priorities for and trade-offs between product/service attributes and to identify the most desired package of attributes (45). DCEs have successfully been applied previously to elicit preferences for home care services (44) and digital interventions (46) in dementia care.

Moreover, future research should further investigate the views and needs of PwD towards CM technology. We have done so in our previous qualitative study (34) which served as an input for the current study. However, in future research, our current survey could be transformed into an interview scheme suitable to administer among PwD.

Lastly, our results have been obtained in the context of CM technology for home-based dementia care. Future research could investigate the extent to which our findings are applicable in the context of informal care for other groups of home-dwelling individuals with chronic conditions.

## 5. Conclusions

Our study shows that Dutch and German informal caregivers of community-dwelling PwD are overall receptive to using CM technology at this point in their life and even moreso at the thought of the cognitive or physical health of their loved declining in the future. In addition, our study provides insight into which sensor types, use scenarios and personal characteristics of informal caregivers should be given more attention to during the development and implementation of CM technology for home-based dementia care. Based on our results, RF-based sensors, light sensors, temperature/humidity sensors, energy meters, and object-tagged sensors are most likely to be accepted by informal caregivers. Moreover, use scenarios for CM technology focused on detecting emergency situations and monitoring of nocturnal wellbeing should be prioritized. Lastly, our study showed several personal characteristics to be associated with CM technology acceptance, including informal caregivers’ gender, relation with care recipient, eHealth literacy, personal innovativeness, usage of safety technology, and their care recipients' type of cognitive impairment and housing situation. Overall, these findings can help to make adequate choices during development and implementation of CM technology for home-based dementia care, thereby increasing the chance of achieving a fit between technology, user, and use context.

## Data Availability

The datasets presented in this article are not readily available because participants did not explicitly state consent to this end. Requests to access the datasets should be directed to the corresponding author (c.wrede@utwente.nl).

## Supplementary material

Data Sheet 1 Main survey materials.

## References

[1] Global status report on the public health response to dementia. Geneva: World Health Organization (2021). ISBN: 978-92-4-003324-5

[2] Pickard L. A growing care gap? The supply of unpaid care for older people by their adult children in England to 2032. Ageing Soc. (2015) 35(1):96–123. 10.1017/S0144686X13000512

[3] Dalgarno EL Gillan V Roberts A Tottie J Britt D Toole C Home care in dementia: the views of informal carers from a co-designed consultation. Dementia. (2021) 20(7):2261–77. 10.1177/1471301221990504 33530737 PMC8564226

[4] OECD. Addressing dementia: the OECD response. In: OECD Health policy studies. Paris: OECD Publishing (2015). 10.1787/9789264231726-en

[5] Collins RN Kishita N. Prevalence of depression and burden among informal care-givers of people with dementia: a meta-analysis. Age Soc. (2019) 40(11):2355–92. 10.1017/S0144686X19000527

[6] Gilhooly KJ Gilhooly ML Sullivan MP McIntyre A Wilson L Harding E A meta-review of stress, coping and interventions in dementia and dementia caregiving. BMC Geriatr. (2016) 16(1):1–8. 10.1186/s12877-016-0280-8.7 27193287 PMC4872341

[7] Pinquart M Sörensen S. Correlates of physical health of informal caregivers: a meta-analysis. J Gerontol B Psychol Sci Soc Sci. (2007) 62(2):126–37. 10.1093/geronb/62.2.P126 17379673

[8] Dutch Ministry of Health, *Welfare and sport (VWS)*. National dementia Strategy 2021–2030 (2020). Available at: https://www.government.nl/documents/publications/2020/11/30/national-dementia-strategy-2021-2030 (Accessed January 10, 2022).

[9] Gaugler JE Zmora R Mitchell LL Finlay J Rosebush CE Nkimbeng M Remote activity monitoring for family caregivers of persons living with dementia: a mixed methods, randomized controlled evaluation. BMC Geriatr. (2021) 21(1):1–6. 10.1186/s12877-021-02634-8 34922475 PMC8684277

[10] Zwierenberg E Nap H Lukkien D Cornelisse L Finnema E Dijkstra A A lifestyle monitoring system to support (in) formal caregivers of people with dementia: analysis of users need, benefits, and concerns. Gerontechnol. (2018) 17(4):194–205. 10.4017/gt.2018.17.4.001.00

[11] Hussain Z Sheng QZ Zhang WE. A review and categorization of techniques on device-free human activity recognition. J Netw Comput Appl. (2020) 167:102738. 10.1016/j.jnca.2020.102738

[12] Sharma N Brinke JK Van Gemert-Pijnen JE Braakman-Jansen LM. Implementation of unobtrusive sensing systems for older adult care: scoping review. JMIR Aging. (2021) 4(4):e27862. 10.2196/27862 34612822 PMC8529483

[13] Mokhtari G Bashi N Zhang Q Nourbakhsh G. Non-wearable human identification sensors for smart home environment: a review. Sens Rev. (2018) 38(3):391–404. 10.1108/SR-07-2017-0140

[14] Alcalá JM Ureña J Hernández Á Gualda D. Assessing human activity in elderly people using non-intrusive load monitoring. Sensors. (2017) 17(2):351. 10.3390/s17020351 28208672 PMC5335959

[15] Ge Y Taha A Shah SA Dashtipour K Zhu S Cooper J Contactless WiFi sensing and monitoring for future healthcare-emerging trends, challenges, and opportunities. IEEE Rev Biomed Eng. (2022) 16:171–91. 10.1109/RBME.2022.3156810 35254990

[16] Brinke JK Chiumento A Havinga P. Personal hygiene monitoring under the shower using wi-fi channel state information. In: Liang RH Chiumento A Pawelczak P Funk M, editors. 1st Workshop on computer human interaction in IoT applications (CHIIot). Delft (2021);2996:4. Available at: https://ceur-ws.org/Vol-2996/paper4.pdf

[17] Liu C Xiong J Cai L Feng L Chen X Fang D. Beyond respiration: contact less sleep sound-activity recognition using RF signals. Proc ACM Interact, Mobile, Wearable Ubiquitous Technol. (2019) 3(3):1–22. 10.1145/3351254

[18] Klavestad S Assres G Fagernes S Grønli T-M. Monitoring activities of daily living using UWB radar technology: a contactless approach. IoT. (2020) 1(2):320–36. 10.3390/iot1020019

[19] Ho A. Are we ready for artificial intelligence health monitoring in elder care? BMC Geriatr. (2020) 20:1–7. 10.1186/s12877-020-01764-9 PMC7504871 32957946

[20] Jaschinski C Ben Allouch S Peters O Cachucho R Van Dijk JA. Acceptance of technologies for aging in place: a conceptual model. J Med Internet Res. (2021) 23(3):e22613. 10.2196/22613 33787505 PMC8047804

[21] Jaschinski C. (2018). *Independent aging with the help of smart technology: investigating the acceptance of ambient assisted living technologies. [Doctoral dissertation]*. University of Twente. 10.3990/1.9789036546348

[22] Davis FD. Perceived usefulness, perceived ease of use, and user acceptance of information technology. MIS Q. (1989) 13(3):319–40. 10.2307/249008

[23] Venkatesh V Morris MG Davis GB Davis FD. User acceptance of information technology: toward a unified view. MIS Q. (2003) 27(3):425–78. 10.2307/30036540

[24] Peek ST Wouters EJ Van Hoof J Luijkx KG Boeije HR Vrijhoef HJ. Factors influencing acceptance of technology for aging in place: a systematic review. Int J Med Inf. (2014) 83(4):235–48. 10.1016/j.ijmedinf.2014.01.004 24529817

[25] Bixter MT Blocker KA Mitzner TL Prakash A Rogers WA. Understanding the use and non-use of social communication technologies by older adults: a qualitative test and extension of the UTAUT model. Gerontechnology. (2019) 18(2):70. 10.4017/2Fgt.2019.18.2.002.00 31754352 PMC6870985

[26] van Gemert-Pijnen JEWC Nijland N van Limburg M Ossebaard HC Kelders SM Eysenbach G A holistic framework to improve the uptake and impact of eHealth technologies. J Med Internet Res. (2011) 13(4):e1672. 10.2196/jmir.1672 PMC3278097 22155738

[27] Thordardottir B Malmgren Fänge A Lethin C Rodriguez Gatta D Chiatti C. Acceptance and use of innovative assistive technologies among people with cognitive impairment and their caregivers: a systematic review. BioMed Res Int. (2019) 2019:18. 10.1155/2019/9196729 PMC6431399 30956989

[28] Guisado-Fernández E Giunti G Mackey LM Blake C Caulfield BM. Factors influencing the adoption of smart health technologies for people with dementia and their informal caregivers: scoping review and design framework. JMIR aging. (2019) 2(1):e12192. 10.2196/12192 31518262 PMC6716546

[29] Holthe T Halvorsrud L Karterud D Hoel KA Lund A. Usability and acceptability of technology for community-dwelling older adults with mild cognitive impairment and dementia: a systematic literature review. Clin Interv Aging. (2018) 13:863–86. 10.2147/CIA.S154717 29765211 PMC5942395

[30] Dequanter S Fobelets M Steenhout I Gagnon MP Bourbonnais A Rahimi S Determinants of technology adoption and continued use among cognitively impaired older adults: a qualitative study. BMC Geriatr. (2022) 22(1):1–6. 10.1186/s12877-022-03048-w 35484488 PMC9047390

[31] Claes V Devriendt E Tournoy J Milisen K. Attitudes and perceptions of adults of 60 years and older towards in-home monitoring of the activities of daily living with contactless sensors: an explorative study. Int J Nurs Stud. (2015) 52(1):134–48. 10.1016/j.ijnurstu.2014.05.010 24951084

[32] Chen K Chan AH. Gerontechnology acceptance by elderly Hong Kong Chinese: a senior technology acceptance model (STAM). Ergonomics. (2014) 57(5):635–52. 10.1080/00140139.2014.895855 24655221

[33] Wrede C Braakman-Jansen A van Gemert-Pijnen L. Requirements for unobtrusive monitoring to support home-based dementia care: qualitative study among formal and informal caregivers. JMIR aging. (2021) 4(2):e26875. 10.2196/26875 33843596 PMC8076981

[34] Wrede C Braakman-Jansen A van Gemert-Pijnen L. How to create value with unobtrusive monitoring technology in home-based dementia care: a multimethod study among key stakeholders. BMC Geriatr. (2022) 22(1):1–9. 10.1186/s12877-022-03550-1 36451119 PMC9713088

[35] Bastoni S Wrede C da Silva MC Sanderman R Gaggioli A Braakman-Jansen A Factors influencing implementation of eHealth technologies to support informal dementia care: umbrella review. JMIR aging. (2021) 4(4):e30841. 10.2196/30841 34623314 PMC8538023

[36] Hvalič-Touzery S Dolničar V Prevodnik K. Factors influencing informal carers’ acceptance of assistive telecare systems in the pre-and post-implementation phase: a scoping study. Health Soc Care Community. (2022) 30(5):e1484–504. 10.1111/hsc.13840 35574935 PMC9541532

[37] Bédard M Molloy DW Squire L Dubois S Lever JA O’Donnell M. The Zarit burden interview: a new short version and screening version. Gerontologist. (2001) 41(5):652–7. 10.1093/geront/41.5.652 11574710

[38] Kayser L Karnoe A Furstrand D Batterham R Christensen KB Elsworth G A multidimensional tool based on the eHealth literacy framework: development and initial validity testing of the eHealth literacy questionnaire (eHLQ). J Med Internet Res. (2018) 20(2):e36. 10.2196/jmir.8371 29434011 PMC5826975

[39] Agarwal R Prasad J. A conceptual and operational definition of personal innovativeness in the domain of information technology. Info Syst Res. (1998) 9(2):204–15. 10.1287/isre.9.2.204

[40] Chapman DW Carter JF. Translation procedures for the cross cultural use of measurement instruments. Educ Eval Policy Anal. (1979) 1(3):71–6. 10.3102/01623737001003071

[41] Kim D Bian H Chang CK Dong L Margrett J. In-home monitoring technology for aging in place: scoping review. Interact J Med Res. (2022) 11(2):e39005. 10.2196/39005 36048502 PMC9478817

[42] Demiris G Oliver DP Giger J Skubic M Rantz M. Older adults’ privacy considerations for vision based recognition methods of eldercare applications. Technol Health Care. (2009) 17(1):41–8. 10.3233/THC-2009-0530 19478404

[43] Berridge C Turner NR Liu L Karras SW Chen A Fredriksen-Goldsen K Advance planning for technology use in dementia care: development, design, and feasibility of a novel self-administered decision-making tool. JMIR Aging. (2022) 5(3):e39335. 10.2196/39335 35896014 PMC9377442

[44] Chester H Clarkson P Davies L Sutcliffe C Davies S Feast A HOST-D (home support in dementia) programme management group. People with dementia and carer preferences for home support services in early-stage dementia. Aging Ment Health. (2018) 22(2):270–9. 10.1080/13607863.2016.1247424 27849124

[45] Kaambwa B Ratcliffe J Shulver W Killington M Taylor A Crotty M Investigating the preferences of older people for telehealth as a new model of health care service delivery: a discrete choice experiment. J Telemed Telecare. (2017) 23(2):301–13. 10.1177/1357633X16637725 26985004

[46] O’Philbin L Woods B Holmes E. People with dementia and caregiver preferences for digital life story work service interventions. A discrete choice experiment and digital survey. Aging Ment Health. (2020) 24(2):353–61. 10.1080/13607863.2018.1525606 30587008

