# Supplementary material for: Understanding acceptance of contactless monitoring technology in home-based dementia care: a cross-sectional survey study among informal caregivers
Source: Front Digit Health. 2023 Oct 4;5:1257009. doi: 10.3389/fdgth.2023.1257009 (PMC10582629; doi:10.3389/fdgth.2023.1257009)
Supplement: Data Sheet 1 — Main survey materials. [file Datasheet1.pdf]

Supplementary Material: Main survey materials

[Note: English non-validated version, translated from the original Dutch/ German version]

1 Introduction to CM technology used in the survey

More and more technologies are being developed that aim to support informal caregivers and enable their loved ones to live at home for longer. In this part of the questionnaire we focus on a special form of assistive technology: Contactless technology for monitoring your care-receiving loved one.
Contactless monitoring technology is a sensor system for the home environment. It is intended as an aid for the informal caregiver to keep an eye on things from the distance. The technology works in a contactless way which means that your loved one does not have to wear any devices (for example, you could think of a radar sensor placed in the corner of the house). Using artificial intelligence, the sensor(s) can learn to recognize your loved one's daily activities and detect important changes, such as nocturnal unrest or a decrease in selfcare. In case of an emergency (such as a fall), the system can alert caregivers. The monitoring information can be displayed on a digital platform (e.g. an app) that is accessible to the informal caregiver and loved one. If desired, the information can also be shared with involved healthcare professionals.

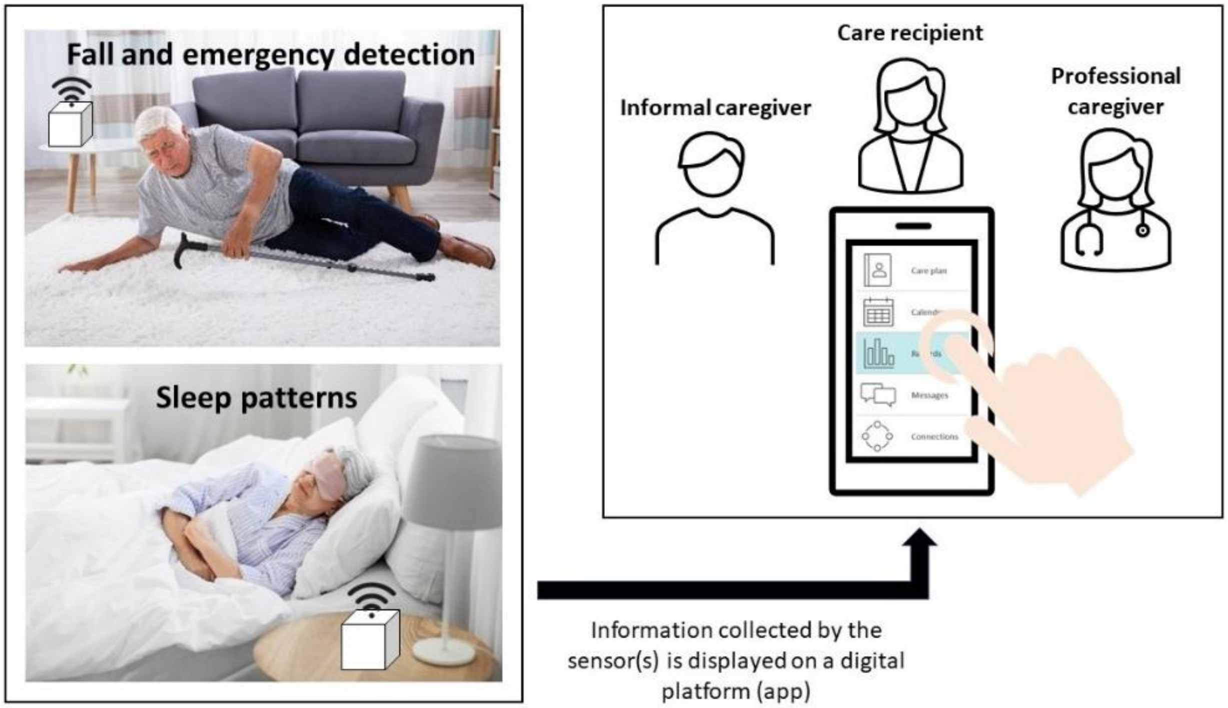

(Figure shown to illustrate the general concept of CM technology)

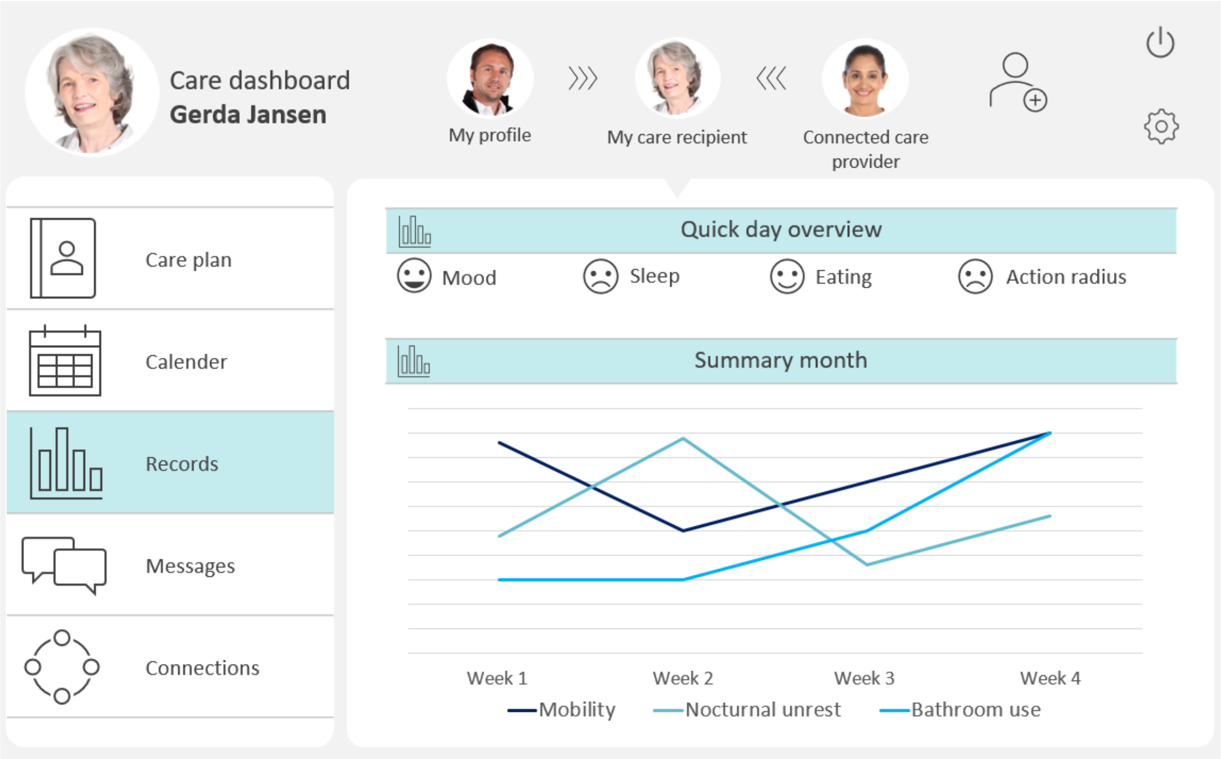

(Page showing a mock-up of a digital platform interface that visualizes possible monitoring information shared within the care network)

2 Survey items: Acceptance towards different contactless sensor types

Contactless monitoring technology can (depending on preference) use different devices to collect information about the situation of your loved one. Please indicate for each of the devices below to what extent you find them acceptable in the care of your home-dwelling loved one.

	Very unacceptable	Unacceptable	Neutral	Acceptable	Very acceptable
1. Devices based on radio frequencies (such as, e.g., a centrally placed sensor that can detect movements within the house via radar)	☐	☐	☐	☐	☐
2. Devices based on sound detection (such as microphones, smart speakers)	☐	☐	☐	☐	☐
3. Visual devices (such as cameras that produce anonymized footage, i.e. images where faces cannot be recognized)	☐	☐	☐	☐	☐

4. Light sensors (sensors that detect whether the light is on or off)	☐	☐	☐	☐	☐
5. Temperature/humidity sensors	☐	☐	☐	☐	☐
6. Energy meters (sensors that monitor the energy consumption of household appliances)	☐	☐	☐	☐	☐
7. Object-tagged devices (sensors attached to objects of daily use such as motion sensors on doors or the fridge, or pressure sensors on bed mattresses)	☐	☐	☐	☐	☐

3 Use scenario descriptions

Scenario 1: Detection of emergency situations
Imagine the following: Contactless monitoring technology has been installed in the home of your loved one. The technology will continuously monitor the safety of your loved one throughout the house. The system can for example detect fall incidents or wandering and can immediately inform you as a caregiver (or another designated person) about this emergency situation.

Scenario 2: Prediction of acute situations
Imagine the following: Contactless monitoring technology has been installed in the home of your loved one. The technology aims not only to detect but to predict acute situations. For example, the technology can continuously monitor the walking speed and walking pattern of your loved one. Using this information, the system can predict your loved one's risk of falling and can inform you (or another designated person) about the situation. The purpose of this is to prevent emergency situations such as falling.

Scenario 3: Monitoring of selfcare behaviors
Imagine the following: Contactless monitoring technology has been installed in the home of your loved one. The technology will continuously monitor the selfcare of your loved one, such as eating, drinking, or personal hygiene (e.g. washing, toileting, dressing). The monitoring system can detect major deviations in selfcare and inform you (or another designated person) about this.

Scenario 4: Monitoring of nocturnal wellbeing
Imagine the following: Contactless monitoring technology has been installed in the home of your loved one. The technology will continuously monitor your loved one's wellbeing throughout the night. The monitoring system can detect deviations from the usual nightly pattern (such as nocturnal unrest, sleeping problems or an unstable day and night rhythm) and can inform you (or another designated person) about this.

Scenario 5: Monitoring of gradual health status changes
Imagine the following: Contactless monitoring technology has been installed in the home of your loved one. The technology will monitor changes that gradually develop in the health of your loved one over a longer period of time. The monitoring system can, for example, inform you (or another designated person) about cognitive or physical changes in your loved one within a certain period.

4 Survey items for each use scenario

Contactless monitoring technology for [scenario X]…	**Totally disagree**	**Disagree**	**Neutral**	**Agree**	**Totally agree**
…is something that I would find acceptable. (A-IC)	☐	☐	☐	☐	☐
…is something that my loved one would find acceptable. (A-CR)	☐	☐	☐	☐	☐
…would be useful to me. (PU1)	☐	☐	☐	☐	☐
…would support me in providing or organizing the best possible care. (PU2)	☐	☐	☐	☐	☐
…would enable me to feel reassured about my loved one's situation. (PU3)	☐	☐	☐	☐	☐
…would help me respond more quickly to my loved one's care needs. (PU4)	☐	☐	☐	☐	☐
…would help me to let my loved one live at home for longer. (PU5)	☐	☐	☐	☐	☐
…would help me to continue as an informal carer for longer. (PU6)	☐	☐	☐	☐	☐
I would like to use contactless monitoring technology for [scenario X] in the near future. (IU)	☐	☐	☐	☐	☐

Legend: A-IC: Acceptability for informal caregiver; A-CR: Acceptability for care recipient; PU: Perceived usefulness; IU: Intention to use
